# Osteogenesis Imperfecta Type 3 in a 10-Year-Old Child With Acute Respiratory Distress Syndrome

**DOI:** 10.7759/cureus.22198

**Published:** 2022-02-14

**Authors:** Delange Augustin, Delange Hendrick Augustin, Daniel David, Jefferson Arnold Théodas, Albertini Fritzlet Derisier

**Affiliations:** 1 Radiology, Hôpital d l'Universite d'Etat d'Haiti (HUEH), Port-au-Prince, HTI; 2 Orthopedics and Traumatology, Hôpital Universitaire la Paix (HUP), Port au Prince, HTI; 3 Pediatric Medicine, Hôpital d l'Universite d'Etat d'Haiti (HUEH), Port-au-Prince, HTI; 4 Orthopedics and Traumatology, Hôpital d l'Universite d'Etat d'Haiti (HUEH), Port-au-Prince, HTI

**Keywords:** 10 years old child, acute respiratory distress syndrome, respiratory failure, osteogenesis imperfecta type 3, oi osteogenesis imperfecta

## Abstract

Osteogenesis imperfecta (OI) represents a group of rare connective tissue disorders characterized by excessive bone fragility. Type 3 is a rare form with new mutations; osteopenia and bone fragility are significant with numerous fractures, continuous and severe deformity of the spine, and long bones. Our case study concerns a 10-year-old male child admitted to the pediatric department of the State University of Haiti Hospital. OI type 3 was diagnosed based on both clinical and radiological assessments. Multidisciplinary care was initiated. Although the evolution was still unsatisfactory, characterized by intermittent episodes of dyspnea and left lung hypoplasia, he was stabilized after 28 days of hospitalization and referred to the orthopedics department for follow-up care.

## Introduction

Osteogenesis imperfecta (OI) represents a group of rare inherited connective tissue disorders characterized by excessive bone fragility [1]. It is caused by genetic mutations in the alpha 1 and alpha 2 chains of type 1 procollagen [2]. Four types were originally described by Sillence in 1979 [2]; type 1 is mild, type 2 is fatal and perinatal, type 3 is severe and characterized by progressive bone deformity, and type 4 is moderately severe.

The overall incidence of OI is roughly one case per 20,000 live births; however, the true prevalence may be higher due to the underdiagnosis of the mild form. The prevalence appears to be alike worldwide, although, there have been more cases recorded in two major tribal groups in Zimbabwe [3]. An autosomal dominant pattern is mainly observed but autosomal recessive forms have also been reported [4,5]. While the diagnosis is mainly clinical, the use of x-rays and confirmation by analysis of the culture of collagen synthesized by the skin fibroblast or blood DNA analysis proves to be necessary in certain cases [6,7].

OI is an incurable disease. The management is multidisciplinary, which mostly required surgery, physiotherapy, and rehabilitation. The medical treatment, particularly based on bisphosphonates, seems promising [6-8]. OI type 3 is a severe, progressive, rare form of autosomal dominant transmission with new mutations. Osteopenia and bone fragility are significant, with numerous fractures, continuous and severe deformity of the spine, and long bones with age. The sclera is normal. The color changes during the prepubertal period, from pale blue or gray at birth to becoming normal during adolescence or adulthood. The skull bone is poorly ossified with the presence of Wormian bones, and also large and thin. Intellectual ability is not affected [8,9].

This case is studied and reported because of its rarity, especially among black people [10], and also, with the aim of reviewing the literature to spotlight the challenges in its management.

## Case presentation

**Figure 1:**
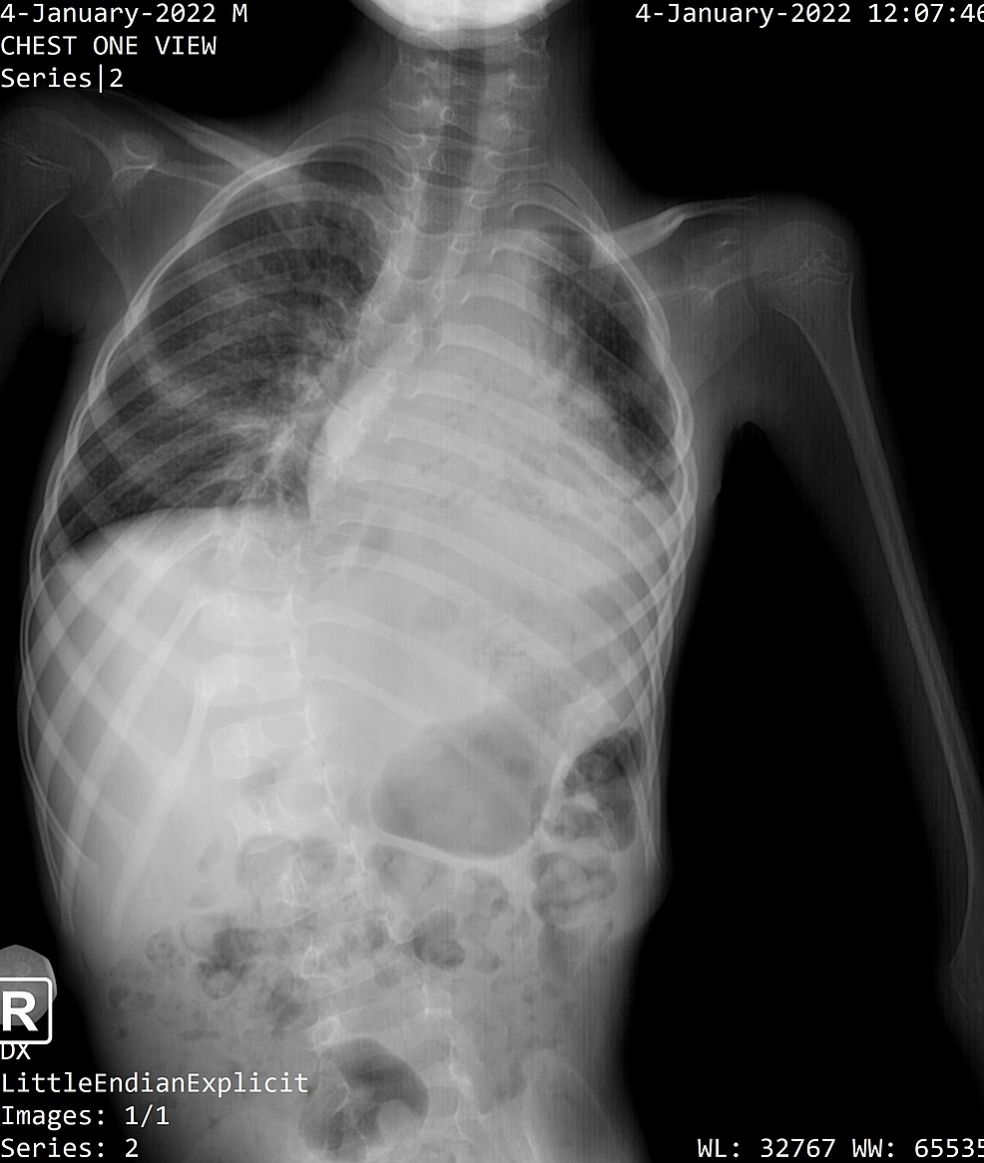
Left pulmonary pneumonia

This case involves a 10-year-old male child with a history of progressive functional limitation of the lower limbs movements and recurrent pneumonia. He was transferred to the State University of Haiti Hospital for fever, dyspnea, digestive disorders, and initial clinical suspicion of acute respiratory distress syndrome on a background of probable Duchenne muscular dystrophy. He was hospitalized, placed on oxygen therapy, and given broad-spectrum antibiotic therapy after chest x-ray results revealed massive neutrophil-predominant left pulmonary pneumonia (Figure 1).

Physical examination revealed normal sclera, inverted triangle face shape, asymmetrical, emaciated thorax with scoliotic deformity of the dorso-lumbar spine, rhonchi sounds in the lungs and decreased vesicular murmurs in the left pulmonary field, and limited upper limbs function associated with loss of lower limbs function since the age of one year after a normal psychomotor development in the neonatal period. Generalized muscle wasting affecting the limbs was also observed. Intellectual ability was preserved.

**Figure 2:**
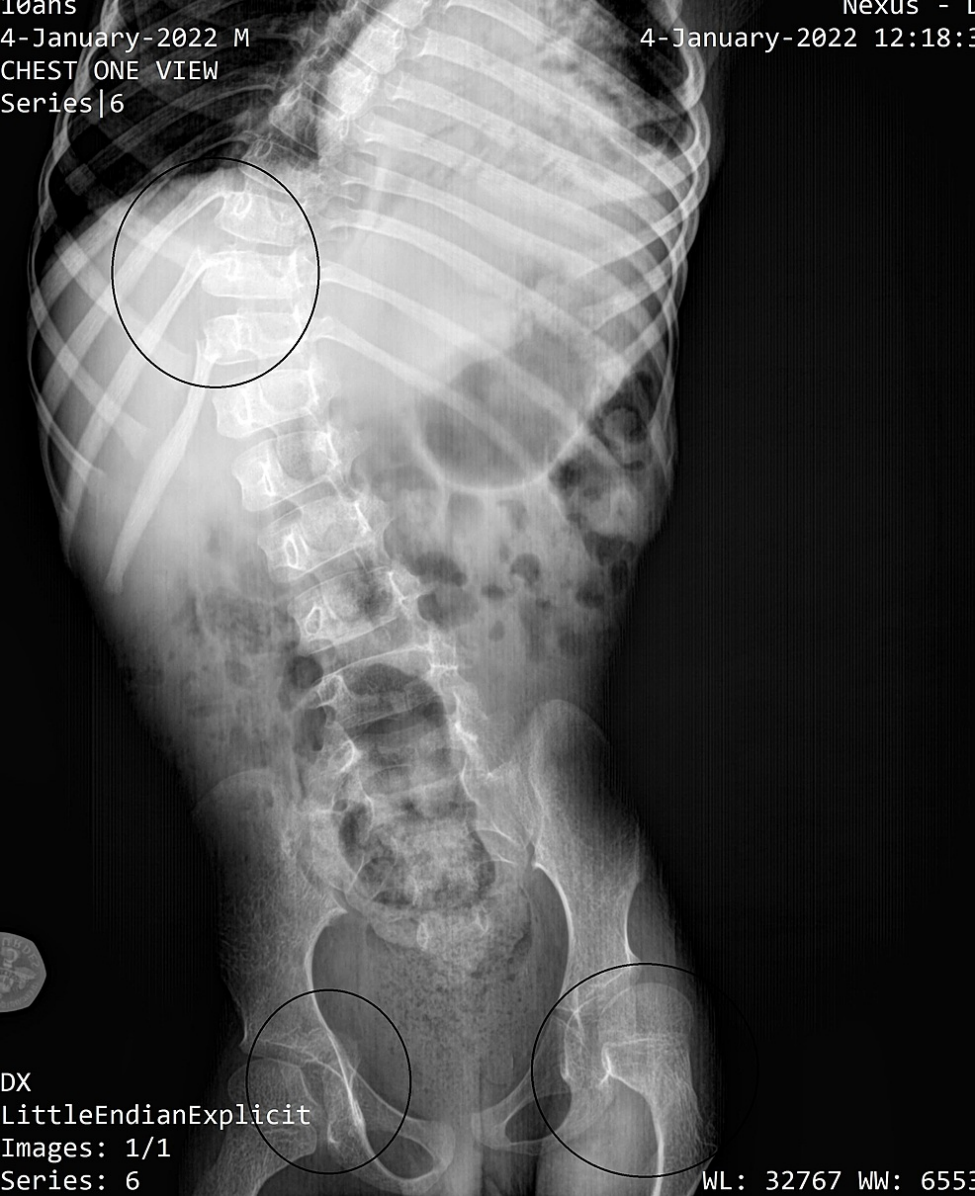
Vertebral column and pelvis (x-ray findings)

The cardiac ultrasound showed mild mitral and tricuspid valve regurgitation with an ejection fraction of 66%. The radiographic assessment revealed kyphoscoliosis with a Cobb angle of 59.57˚, fractures of the 10th, 11th, and 12th right ribs, protrusion of the acetabular roof projecting toward the pelvis, fracture of the right femoral head, opacification of the growth plate of the left femoral head associated with coxa magna, and neglected intertrochanteric greenstick fracture. It was concluded as progressive OI type 3, in correlation with the clinical history (Figure 2).

Although the evolution was still unsatisfactory, characterized by intermittent episodes of dyspnea and left lung hypoplasia, he was stabilized after 28 days of hospitalization and referred to the orthopedics department for follow-up care.

## Discussion

The clinical picture of OI is mainly characterized by skeletal manifestations. The radiological findings in our patient revealed kyphoscoliosis with a Cobb angle of 59.57˚, fractures of the 10th, 11th and 12th ribs, protrusion of the right acetabular roof toward the pelvis, left intertrochanteric greenstick fracture associated with opacification of the growth plate of the femoral head, and coxa magna of the corresponding bone. Our diagnostic approach was mainly based on these findings. Indeed, type 3 is a severe and progressive form. Bone fragility and osteopenia are significant and progress with age, manifesting by numerous fractures, severe and continuous deformity of the long bones and spine [9]. Physical examination revealed normal sclera, inverted triangle face shape. In OI type 3, the sclera, which is pale blue or gray at birth, changes during childhood and prepubertal period, becoming normal with age [9].

The child was admitted with a clinical picture of acute respiratory distress syndrome; however, he still presented with intermittent dyspnea, even after ceasing to be a medical emergency. OI patients with scoliosis have a progressive decline of forced vital capacity (FVC), total lung capacity (TLC), and vital capacity (VC), directly proportional to the worsening of scoliosis. Normal lung function parameters drop sharply in patients with Cobb angle greater than 30˚; for greater curvature, these parameters will continue to drop progressively [11].

The chest x-ray on admission showed massive left pneumonia, with neutrophils predominance in the white blood cell count. The clinical picture of OI type 3 and the imaging follow-ups challenged us on a key aspect of radiology, the As Low, As Reasonably Achievable (ALARA) principle. Indeed, OI is an autosomal dominant disease with new mutations [9]. X-ray exposure can produce genetic mutations [12]. Minimizing the x-ray exposure of OI type 3 patients may prove very beneficial. Using chest ultrasound instead of chest x-ray as a routine examination might be a better alternative for assessing respiratory infections. Although chest x-rays are the most commonly used means of diagnosing pneumonia [13], it has a sensitivity of less than 75% compared to the Ct-scan [14-16]. And even if the CT-scan has a high sensitivity for diagnosing pneumonia, it is not appropriate for routine investigations [13]. There is a growing body of evidence-based research on the use of ultrasound for diagnosing pneumonia, with a sensitivity around 89-97% and a specificity between 95% and 98% [17-26]. A scientific journal suggests that the ultrasound could even replace the CT-scan for diagnosing and monitoring patients with acute respiratory distress and common complications such as pleurisy and pneumothorax [17].

The follow-up chest x-ray, after antibiotic therapy, found left lung hypoplasia. Although skeletal findings are predominant in OI, this is a generalized connective tissue disease affecting the type I collagen, which, in addition to bone, skin, and tendons [27], constitutes nearly 80% of the lung and heart parenchyma [11]. The non-skeletal respiratory and cardiovascular manifestations have a high rate of morbidity and mortality in severe and moderate forms of OI, but the latter has always been considered manifestations secondary to skeletal changes and not to the underlying mutation of type I collagen [28,29]. The pulmonary complications represent the leading cause of death in people suffering from OI; usually considered secondary to scoliosis or rib fractures [7,29-31], two case studies of fatal OI with severe pulmonary hypoplasia suggest that collagen damage may be the primary cause of pulmonary complications [32,33].

Chest x-ray is the first-line radiology assessment for respiratory infections at the State University of Haiti Hospital; however, once the diagnosis of OI type 3 was made, priority was given to lung ultrasound for any subsequent pneumonia. Indeed, more clinicians should be made aware of advances regarding the use of pulmonary ultrasound in diagnostic procedures and the management of respiratory infections, among other things, its importance in the management of OI type 3.

The cardiac ultrasound showed mild mitral and tricuspid valve regurgitation with an ejection fraction of 66%. Cardiovascular manifestations in the form of valvular heart disease, aortic dilatation, atrial septal defect, and septal and left ventricular posterior wall thickening have been reported in several cases of OI including type 3 [11,34-38]. A study conducted by Thiele et al. identified five OI type 3 patients with impaired cardiac function on the ECG (sinus tachycardia, sinus tachycardia with Q wave, etc.) [11].

Surgical management has long been the main type of intervention in OI patients, along with physiotherapy and rehabilitation. However, the persistence of bone fragility with recurrent fractures has led to the search for alternative treatment, mainly medication, with the main objective of strengthening the bone structure [10].

Currently, bisphosphonate is the only medicine licensed specifically to manage OI [3]. They are similar to inorganic pyrophosphate and act by binding to hydroxyapatite in the bone matrix, thus hindering crystal dissolution. They put a stop to the attachment of osteoclasts to the bone matrix and the recruitment and survival of osteoclasts, thus preventing bone resorption, which in turn expand bone density and strength [3,10].

Since first published in 1987, many researchers have tried to evaluate the use of bisphosphonates in the treatment of OI using distinct treatment regimens, with several publications. Pamidronate administered in intravenous infusion cycles appears to be the preferred treatment for children with OI. A lot of researchers have mentioned an expansion of bone density along with a reduction in the risk of fracture [39-41]. Risedronate has been proposed as a second-line treatment due to its lower bone mineral affinity than nitrogen-containing bisphosphonates, such as alendronate and olpadronate [42]. Antoniazzi et al. also demonstrated that administrating bisphosphonate along with growth hormone is a more effective treatment than the use of bisphosphonates alone [43].

However, despite the fact that various researchers have published their results showing enhancement of linear growth, bone mineral density, reduction of fracture rate, and chronic bone pain, the fundamental goal of the treatment has not yet been achieved. Concerns revolve around the duration of the treatment until reaching an expected result, the effects of prolonged use of bisphosphonates in children [7,44,45], and the time necessary for fractures’ consolidation after osteotomies [46]. Rauch et al. observed a minimal benefit of bisphosphonates after two to four years of therapy [46].

Bone marrow transplantation has been assessed with a documented improvement in the reduction of the fracture rate, increase in bone density, and linear bone growth, this latter is partly related to the increase in the collagen level in proportion to the increase in the number of osteoblasts after transplantation [10,47].

There is no doubt that medical treatment has improved the management of children suffering from OI. Ultimately, OI should be cured by eliminating the genetic mutation and genetic therapy is being investigated as a potential upcoming treatment of the OI. Until then, palliative treatment is the only option [10,48].

Due to recurrent pulmonary infections, the OI Foundation [49] highly recommends coronavirus disease 2010 (COVID-19) vaccination in all people living with OI and having no contraindications, and also recommends to all people living with OI to: avoid active and passive smoking, consult a physician in case of respiratory infection, maintain adequate hydration, keep the upper body strong by exercises aimed at strengthening the chest muscles to improve lung capacity; get vaccinated against flu and pneumonia, and regularly monitor lung function and oxygen saturation with a pulse oximeter [50]. Sometimes, respiratory machines, such as continuous positive airway pressure (CPAP) or bilevel positive airway pressure (BiPAP), can be made available to the patient in order to improve lung function.

## Conclusions

OI type 3 is a rare hereditary disease. This case study presents one of the rare cases recorded in Haiti. Our literature review highlighted the challenges faced by hospitals with inadequate infrastructure, especially in the absence of a surgeon specializing in spine surgery, and also the latest advances in the management of these patients. Due to the possibility of new mutations in OI type 3, the question is how to limit these patients’ exposure to x-rays, knowing that exposure to x-rays alone can lead to new genetic mutations. The use of lung ultrasound for assessing respiratory infections has been proposed as an alternative to chest x-ray, thus solving this new concern.
